# Supplementary material for: Genetic regulation of post-translational modification of two distinct proteins
Source: Nat Commun. 2022 Mar 24;13:1586. doi: 10.1038/s41467-022-29189-5 (PMC8948205; doi:10.1038/s41467-022-29189-5)
Supplement: Supplementary file 1 — Supplementary Information [file 41467_2022_29189_MOESM1_ESM.pdf]

Same role but different actors genetically regulating the post-translational modification of two distinct proteins

Landini et al.

Supplementary Methods

Transferrin isolation

Flowthrough during IgG purification was collected for immediate subsequent Tf isolation using previously developed preconditioned CIMac-@Tf 96-well monolithic plate[1]. Unbound proteins during Tf isolation were washed away with 1x PBS (0.25 mol L^{-1} NaCl), pH 7.4. Bound Tf was eluted with 0.7 mL of 0.1 mol L^{-1} formic acid pH 3.0 (pH adjusted with 25 % ammonia solution, Merck) and immediately neutralized with 1 mol L^{-1} ammonium hydrogencarbonate (Sigma-Aldrich) to pH 7.0. Monolithic plate was regenerated and stored at 4 °C until the next isolation. Each elution fraction (300 µL) was dried in a vacuum centrifuge (Thermo Scientific) and stored at -20 °C until subsequent N-glycan release. To ensure quality of transferrin purification from isolation to isolation we randomly selected transferrin eluates (7.5x concentrated) per each plate and analysed them by SDS-PAGE to check for potential contaminants. As can be seen in the Supplementary Figure 11, the purification was successful and no other contaminants were detected. We next analysed transferrin eluate for transferrin purity by performing trypsin digestion and LC-MS analysis of obtained (glyco)peptides. Pooled isolated transferrin sample was reduced with dithiothreitol and alkylated with iodoacetamide prior to trypsin digestion. Tryptic glycopeptides and peptides were separated and analysed by nano liquid chromatography coupled to electrospray ionization quadrupole time of flight mass spectrometry (nanoLC-ESI-qTOF-MS). A search for specific tryptic peptides with a maximum of 2 miscleavages was done in MaxQuant software[2] against *Homo sapiens* proteins sequences (UniProt fasta file) with the methionine oxidation and asparagine carrying *N*-acetylhexosamine as variable modifications and carbamidomethyl on cysteine as the fixed modification. Analysis was performed in triplicates and the average intensity extracted for serotransferrin (UniProt P02787) was 99.36%, which confirms high purity of the transferrin sample.

N-glycan release and fluorescent labelling

Dried Tf eluates were denatured with 30 µl of 13.3 g L^{-1} sodium dodecyl sulfate (SDS, Invitrogen) and by incubation at 65 °C for 10 min. After cooling down to room temperature for 30 min, 10 µl of 4 % (v/v) Igepal CA-630 (Sigma-Aldrich) was added and the mixture was shaken for 15 min on a plate shaker. N-glycans were released after the addition of 10 µL of 5x PBS and 1.2 U of PNGase F (Promega) by incubation at 37 °C for 18 hours. Released N-glycans were labeled with 2-aminobenzamide (2-AB, Sigma-Aldrich). The labeling mixture was freshly prepared by dissolving 2-AB and 2-methylpyridine borane complex (2-PB, Sigma-Aldrich) (final concentrations of 19.2 mg mL^{-1} and 44.8 mg mL^{-1}, respectively) in the mixture of dimethyl sulfoxide (Sigma-Aldrich) and glacial acetic acid (Merck) (7:3). Labeling mixture (25 µL) was added to each sample and the plate was sealed using an adhesive seal. After 10 minutes of shaking, samples were incubated for 2 hours at 65 °C. Excess of reagents from previous steps was removed from the samples using hydrophilic interaction liquid

chromatography solid phase extraction (HILIC-SPE). After free N-glycan labeling samples were cooled down to room temperature for 30 min and 700 μL of acetonitrile (previously cooled down to 4 °C) was added to each sample. The cleanup procedure was performed on a hydrophilic 0.2 μm AcroPrep GHP filter plate (Pall) using a vacuum manifold (Pall) at around 25 mm Hg. All wells of a GHP filter plate were prewashed with 200 μL of 70 % (v/v) ethanol in water, 200 μL of ultrapure water, and 200 μL of 96 % (v/v) acetonitrile in water (previously cooled down to 4 °C). Diluted samples were loaded to the GHP filter plate wells, and after short incubation subsequently washed with 5x 200 μL of 96 % (v/v) acetonitrile in water. The last washing step was followed by centrifugation at 164 *g* for 5 minutes. Glycans were eluted from the plate with 2x 90 μL of ultrapure water after 15 min shaking at room temperature and centrifugation at 164 *g* for 5 minutes in each step. Combined eluates of 2-AB labeled Tf N-glycans were stored at -20 °C until ultra-high-performance liquid chromatography (UHPLC) analysis.

Glycan analysis by ultra-high-performance liquid chromatography

Fluorescently labelled and purified Tf N-glycans were analyzed by UHPLC based on hydrophilic interactions (HILIC-UHPLC) and detected using excitation and emission wavelengths of 250 and 428 nm, respectively. Acquity UHPLC instrument (Waters) was under the control of Empower 3 software, build 3471 (Waters). Mobile phases were 100 mmol L^{-1} ammonium formate, pH 4.4 (solvent A) and acetonitrile (solvent B) and samples were maintained at 10 °C before injection. Tf 2-AB labeled N-glycans prepared in 75 % acetonitrile were separated on a Waters BEH Glycan column, 150 × 2.1 mm i.d., 1.7 μm BEH particles at 25 °C in a linear gradient of 30-47 % solvent A at a flow rate of 0.56 mL min^{-1} during a 23 minute analytical run. The HILIC-UHPLC system was calibrated using a dextran ladder (external standard of hydrolysed and 2-AB labelled glucose oligomers) according to which the retention times for the individual chromatographic peaks (representing the 2-AB labeled glycan) were converted to glucose units (GU). Data processing was performed using an automatic processing method with a traditional integration algorithm. Each Tf N-glycans chromatogram integrated into 35 peaks was manually corrected to maintain the same intervals of integration for all the samples. The amount of glycans in each chromatographic peak was expressed as a percentage of the total integrated area (% Area).

Replication of transferrin N-glycans loci

To assess robustness of our findings we used the VIKING cohort as replication cohort and CROATIA-Korcula as discovery cohort. Each significant sentinel SNP-top glycan trait pair from the discovery cohort was tested for associations in the replication cohort, with replication significance threshold set to the p-value ≤ 0.00625 (0.05/8, number of discovery cohort genome-wide significant loci). Where the SNP of interest was not available in the replication cohort, a proxy SNP in high linkage disequilibrium (r2 ≥ 0.8) was used instead. In addition to

statistical significance, we also assessed if the direction of estimated effect was concordant between discovery and replication study.

Supplementary Results

Transferrin N-glycans shared genetic associations with complex traits and diseases

For the shared associations between transferrin glycosylation from the *ST3GAL4* locus and LDL, total cholesterol levels and platelet-related traits; *HNF1A* and coronary artery disease, levels of C-reactive protein and of gamma-glutamyl transferase; *FUT6* and age-related macular degeneration (Supplementary Table 11a); the SMR p-value was not significant (Supplementary Table 11b), so the inference on colocalisation could not be performed.

Colocalisation analysis of transferrin and IgG glycan traits with multiple independent association signals at genomic region

To investigate whether the same variant within the *FUT8* and *FUT6* loci is regulating glycosylation of both proteins, and, at the same time, account for the presence of multiple conditionally distinct association signals within the same locus, we applied the PwCoCo pipeline[3], integrating Approximate Bayes Factor (ABF) colocalisation[4] and conditional analyses (for details see Supplementary Figure 1). Briefly, to address the problem of multiple associations within a locus, this approach tests for colocalisation using not only the trait's unconditioned GWAS association statistics, but also their conditioned ones, assessing if any of the independent associations colocalise[3]. We first tested for evidence of multiple SNPs independently contributing to IgG glycan levels at the *FUT6* and *FUT8* loci. While no secondary associated variants were observed in the *FUT6* locus using GCTA-COJO stepwise analysis, two independent variants are likely to contribute to variation in transferrin and IgG glycan traits, namely transferrin TfGP32 and IgG GP20, in the *FUT8* locus (Supplementary Table 5). In this case, colocalisation analyses were conducted between full unconditioned association statistics, association statistics conditioned by one association signal (i.e. transferrin TfGP20 conditioned on rs72716459 and IgG GP7 conditioned on rs8022094) and those conditioned by the other association signal (i.e. transferrin TfGP20 conditioned on rs2411815 and IgG GP7 conditioned on rs8006608), for a total of nine pairwise combinations. We obtained robust evidence against the colocalisation hypothesis for all tested traits, except for transferrin TfGP20 conditioned on rs72716459 and IgG GP7 unconditioned association statistics. In this case in fact it was not possible to strongly support either the hypothesis of different causal variants at the locus or trait colocalisation (PP.H3 = 46.82%, PP.H4 = 53.18%) and therefore whether this glycans pair share genetic architecture at this locus remains unclear (Supplementary Table 15, Supplementary Figure 7-9). It is important to note that another transferrin glycosylation associated genetic region, harbouring *NXEP1* and *NXEP4* genes, was also associated with IgG glycosylation in Klaric *et al.*[5] Since the role of these genes is unknown

and therefore interpretation of their role in glycosylation is not straightforward, we did not proceed with colocalisation analysis for this region.

Colocalisation of TfGP32 and plasma glycosylation traits containing antennary fucose

Transferrin is one of the most abundant proteins in plasma and it can be expected it contributes to plasma glycosylation glycan peaks. To assess whether TfGP32 is likely to have antennary fucose we performed colocalisation analysis of TfGP32 and two plasma glycosylation traits containing antennary fucose (PGP32 - A4F1G3S[3,3+6,3+6]3 and PGP36 - A4F1G4S[3,3,3,6]4) from Sharapov *et al*[6]. and a plasma glycosylation trait reflecting total antennary fucosylation (A-FUC) from Huffman *et al.*[7] By using the approximate Bayes factor colocalisation analysis implemented in coloc R package[4] using the default priors (10^{-4} for p1 and p2 and 10^{-5} for p12), we obtained robust evidence supporting the hypothesis that plasma-derived antennary fucosylation traits colocalise with the TfGP32 (Supplementary Figure 4).

Effect of protein levels on glycosylation

Glycosylation was analysed by releasing total N-glycans from the isolated protein and each glycan structure was quantified as the percentage of the total IgG N-glycome or total transferrin N-glycome. With this approach, only changes in glycosylation are detected (relative abundance of individual glycan species in relation to the whole IgG or transferrin glycome) and not the absolute amounts of specific glycans, which would be affected by changes in protein levels. Unfortunately, abundance of transferrin and IgG was not measured in this study and, consequently, the relation between protein abundance and N-glycan measurements could not be directly investigated. In the case of IgG, we checked whether the specific protocols used for glycan analysis have a bias depending on the initial, already isolated, protein amount. The protocol we used was robust in measuring very similar levels of glycan traits for different IgG abundance (Supplementary Figure 12). In addition, Bermingham *et al.*[8] reported that, while the IgG N-glycan profile varied with IgG levels, adjusting for IgG levels in the analyses made no meaningful difference to associations of glycans with markers of glycaemic control.

To assess the potential impact of transferrin protein levels on transferrin glycome associations we used transferrin cis-protein QTL (pQTL) rs8177240 (LD R^2= 0.02 with the glyQTL rs6785596), the strongest association with transferrin protein levels reported in GWAS catalog (p-value = 8×10^{-610})[9], as a proxy for TF abundance. Interestingly, the pQTL is not an expression QTL (eQTL), but rather a splicing QTL for transferrin levels in liver (p=5.9×10^{-25}, GTEx v8). We then tested its association with transferrin glycans and assessed whether the glycan associations with variants from the *TF* region are likely to be driven by this variant. We considered four models:

M0: glycan ~ age + sex

M1: glycan ~ age + sex + pQTL (rs8177240)

M2: glycan ~ age + sex + glyQTL (rs6785596)

M3: glycan ~ age + sex + pQTL (rs6785596) + glyQTL (rs8177240)

and performed likelihood ratio test between:

- M0 and M1 to assess associations of glycans and pQTL (rs8177240)
- M1 and M3 to assess whether glyQTL contributes to glycan levels even when the pQTL is included in the model
- M2 and M3 to assess whether pQTL contributes to glycan levels even when the glyQTL is included in the model

To control for increased levels of relatedness between subjects in our studies, the models were fitted using linear mixed models as implemented in the lme4qtl R package[10], with age, sex, pQTL and glyQTL as fixed effects and kinship matrix as a random effect. The kinship matrix was estimated from the genotyped data using the "ibs" function from GenABEL[11] R package.

Two glycan traits were significantly ($P \leq 0.05/35 = 1.4\times10^{-3}$) associated with the pQTL. For one of the two traits, TfGP3, the glyQTL contributes to glycan levels in addition to the pQTL, while for the TfGP9 no additional variation is explained by the glyQTL (Supplementary Table 6). To further corroborate these findings we also repeated the meta-analysis conditioning on the transferrin pQTL rs8177240[9], using the conditional approach implemented in the GCTA-COJO "–cond", and genotypes of 10,000 unrelated individuals of white British ancestry from UK Biobank[12] as independent LD reference panel. The only glycan trait that showed a relevant change in effect size and significance of its association was TfGP9 (Supplementary Table 19), suggesting that its association was dependent on the transferrin protein levels. In case of two glycan traits, TfGP3 and TfGP8, the associations were somewhat less significant, but the effect sizes remained very similar. Accordingly, we consider that transferrin protein levels are likely not affecting associations with 2 out of 3 transferrin glycan traits associated with variants from the *TF* gene. This is in accordance with findings from Kutalik *et al.*[13], who used the same approach to show that associations of a-/disialylated transferrin with the *TF* region were independent of associations with transferrin pQTL.

The sentinel glycosylation variant, rs6785596, is a cis eQTL in adipose tissue (Supplementary Table 8a) and it colocalises with TfGP3, but it does not colocalise with expression of *TF* in blood (eQTLGen, Supplementary Figure 13). However, as outlined in the main text, transferrin is predominantly expressed in liver, for which there are no robust transferrin eQTLs (the strongest eQTL in GTEx v8 rs60770862, $p=3.3\times10^{-6}$, LD with glyQTL rs6785596 = 0.0001). The glyQTL variant rs6785596 is also in middling LD (0.57) with a missense variant rs1799899. Overall, further analyses are needed to unravel the complex mechanism behind the associations of transferrin glycans and variants from the *TF* region.

Supplementary Figures

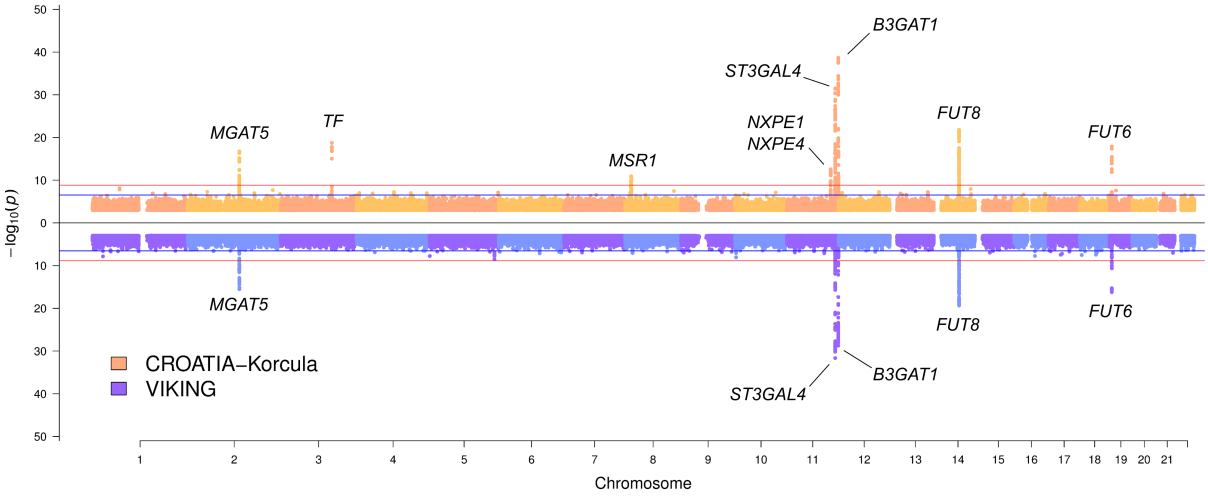

Supplementary Figure 1. Transferrin N-glycome CROATIA-Korcula and VIKING cohorts GWAS summary Miami plot. Miami plot pooling together individual cohort GWAS results obtained across all 35 transferrin glycan traits, at the top in orange for CROATIA-Korcula cohort and at the bottom in blue for VIKING cohort. For each SNP, the lowest p-value across the 35 traits is reported. The y axis shows the strength of the association, and the x axis the genomic position of the SNP. P-values are derived from two-sided Wald test with one degree of freedom. The horizontal red line corresponds to the multiple testing corrected genome-wide significance threshold of 1.43×10^{-9}. The horizontal blue line corresponds to the multiple tests corrected genome-wide suggestive threshold of 2.86×10^{-7}. For simplicity, SNPs with p-value > 1×10^{-3} are not reported. GWAS effect size, standard error and p-value of each sentinel SNP are available in Supplementary Table 1 for CROATIA-Korcula, and in Supplementary Table 2 for VIKING cohort.

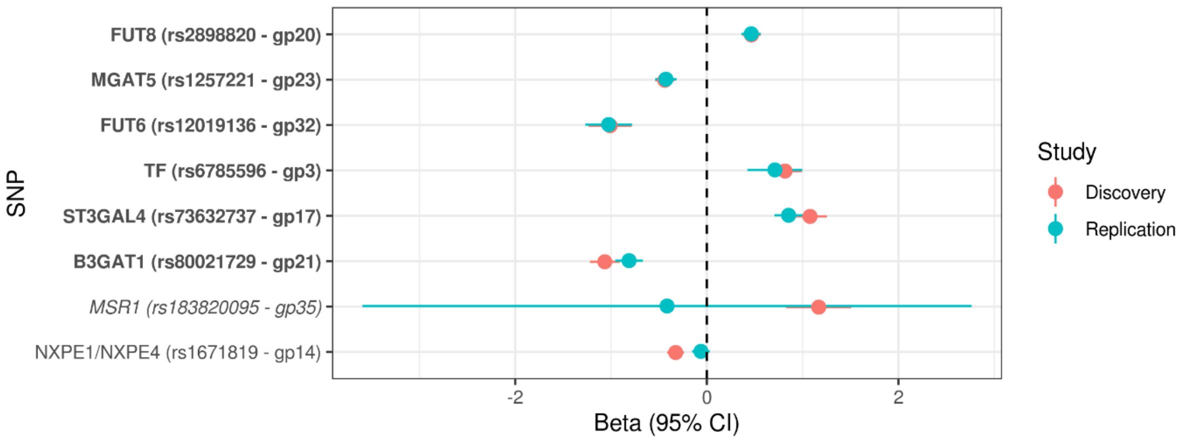

Supplementary Figure 2. Replication of transferrin N-glycome discovery GWAS. Each locus is represented by the strongest SNP-transferrin glycan (reported in brackets) association in the region (y axis). The x axis shows the GWAS effect estimate, with lines representing 95% confidence intervals (CI). For each locus significantly associated with transferrin N-glycome in CROATIA-Korcula cohort, effect size and CI of the sentinel SNP are reported in red when estimated in CROATIA-Korcula cohort (Discovery), and in blue when estimated in VIKING cohort (Replication). Gene names have been marked by different fonts based on overlap between confidence intervals of effect estimates. **In bold:** nominal replication ($p < 0.05/8 = 6.3\times10^{-3}$). *In italics:* CIs overlap and cover zero, and replication estimate is closer to zero than discovery. In roman: CIs do not overlap and replication estimate covers zero. Proxy SNP rs554715390 was used for replicating SNP rs183820095 (D'=1, r^2=1). Effect sizes and their standard errors are derived from two-sided linear regression, P-values are derived from two-sided Wald test with one degree of freedom. GWAS effect size, standard error and p-value of each sentinel SNP are reported in Supplementary Table 1 for discovery, and in Supplementary Table 2 for replication cohort.

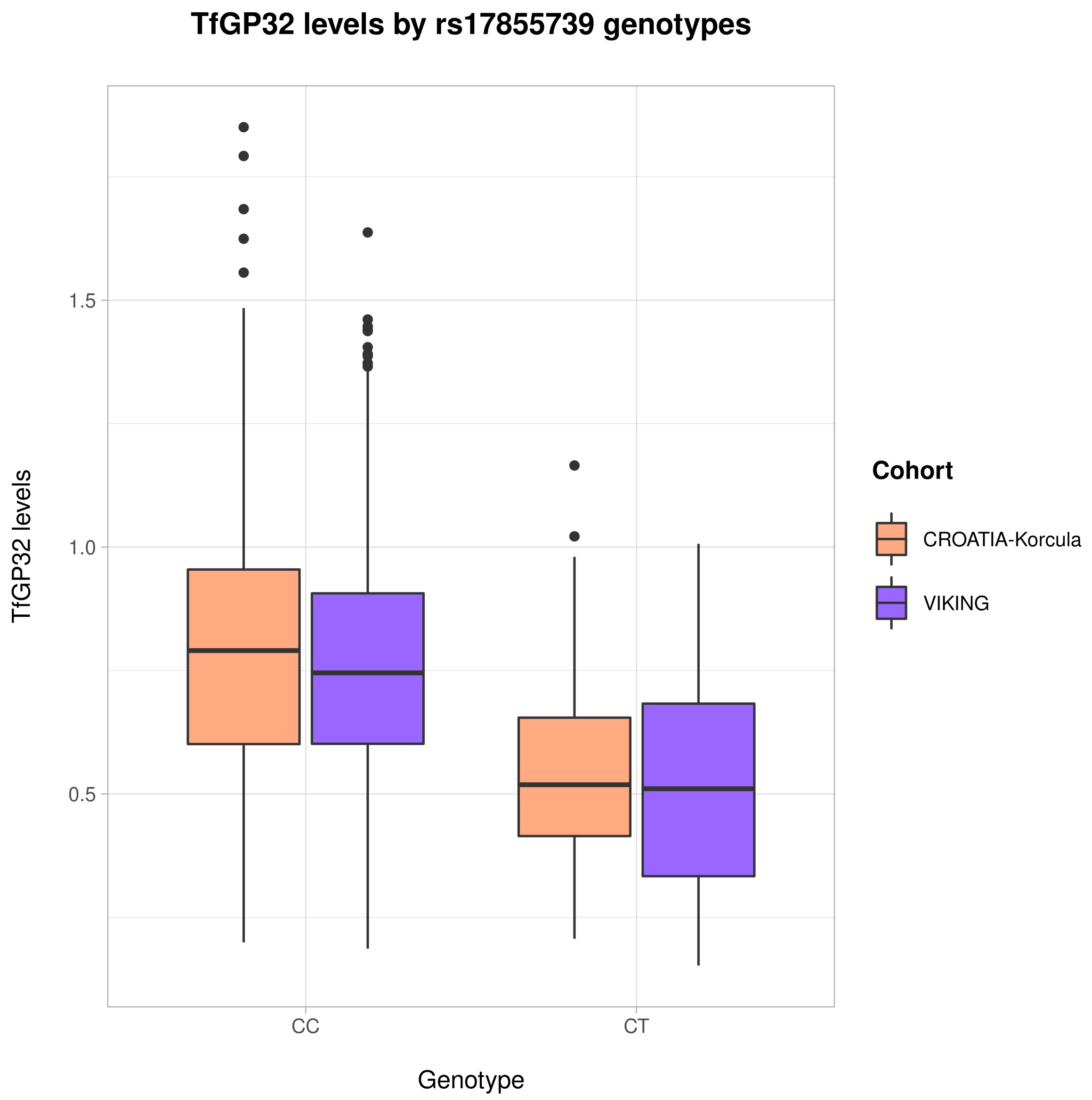

Supplementary Figure 3. **Difference in levels of (normalised and batch corrected) TfGP32 glycan trait by rs17855739 genotypes.** We grouped CROATIA-Korcula (CC genotype N= 859, CT genotype N = 79, TT genotype N = 0) and VIKING (CC genotype N= 890, CT genotype N = 65, TT genotype N = 0) samples based on their genotype at rs17855739 missense variant. rs17855739 is significantly association with TfGP32 glycan trait levels in both CROATIA-Korcula (GWAS p-value=$4.54x10^{-18}$) and VIKING (GWAS p-value = $2.00x10^{-16}$) cohorts. As showed in the boxplot, the median levels of TfGP32 were lower for individuals heterozygous at rs17855739, compared to those having two C alleles. These results suggest that rs17855739 missense mutation significantly decreases the levels of TfGP32, which we suggest as a potential proxy of the activity of alpha-(1,3)-fucosyltransferase 6 enzyme (see Supplementary Results). In the plot, the middle line represents the median, lower and upper limits of the box represent 1st and 3rd quartile, whiskers represent 1.5 interquartile range, points represent individuals with TfGP32 levels that are more than 1.5 interquartile distance away from the 3rd quartile.

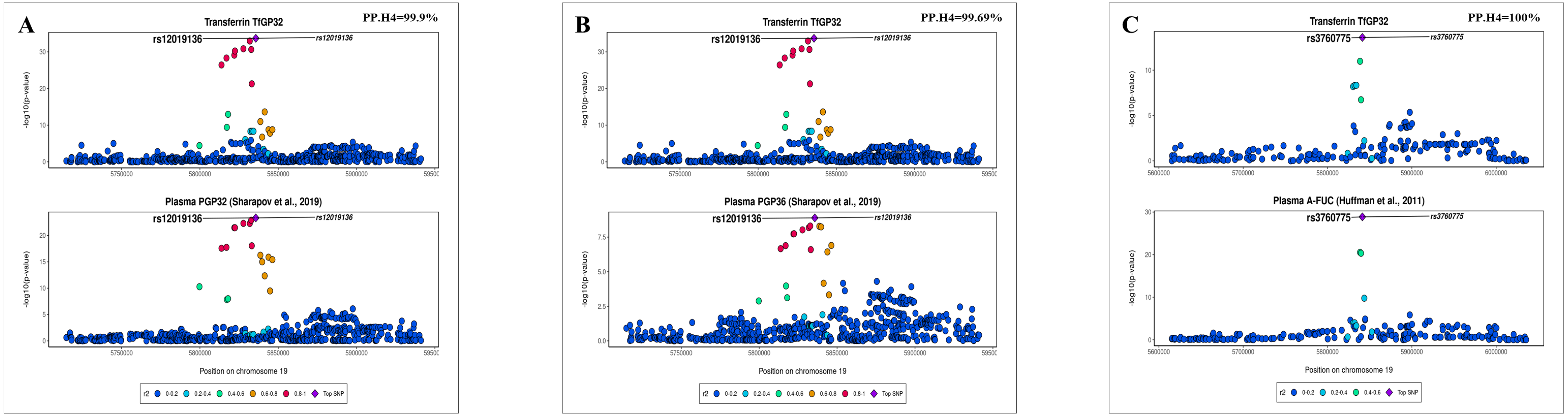

Supplementary Figure 4. Local association patterns of transferrin N-glycan TfGP32 and plasma glycosylation traits related to antennary fucosylation. A) Colocalisation of TfGP32 and total plasma glycan PGP32 - A4F1G3S[3,3+6,3+6]3, PP.H4 = 99.9% **B)** Colocalisation of TfPG32 and total plasma glycan PGP36 - A4F1G4S[3,3,3,6]4, PP.H4 = 99.69% **C)** Colocalisation of TfGP32 and total plasma antennary fucosylation (A-FUC), PP.H4 = 100%. Plasma glycosylation traits containing antennary fucose (PGP32 and PGP36) were taken from Sharapov *et al*[6]. and plasma glycosylation trait reflecting total antennary fucosylation (A-FUC) from Huffman *et al*.[7] For each pairwise colocalisation test, the hypothesis having the higher posterior probability is reported at the top right of the plot. PP.H4 – colocalisation: the two traits are regulated by the same underlying causal variant.

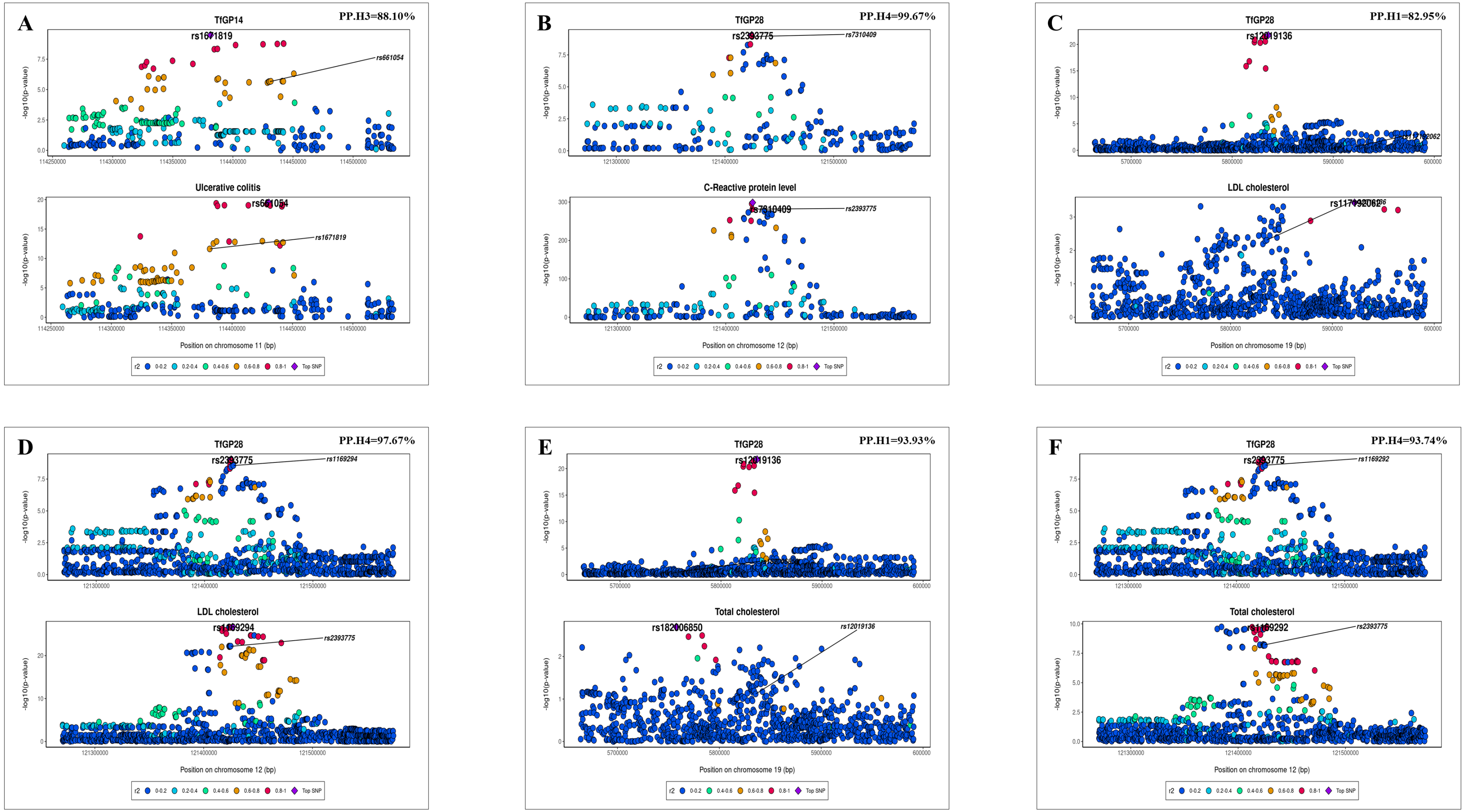

Supplementary Figure 5. Local association patterns of transferrin N-glycans tested for trait colocalisation with complex human traits. GWAS summary statistics of complex traits and diseases were taken from publicly available studies detailed at Supplementary Table 18. **A)** TfGP14 and Ulcerative colitis, in the *NXEP1/NXEP4* locus. PP.H3 = 88.1% **B)** TfGP28 and CRP, in the *HNF1A* locus. PP.H4 = 99.7% **C)** TfGP28 and LDL cholesterol, in the *FUT6* locus. PP.H1 = 83.0% **D)** TfGP28 and LDL, in the *HNF1A* locus. PP.H4 = 97.7% **E)** TfGP28 and total cholesterol, in the *FUT6* locus, PP.H1 = 93.9% **F)** TfGP28 and total cholesterol, in the *HNF1A* locus. PP.H4 = 93.7%. The full colocalisation analysis results can be found at Supplementary Table 13. PP.H1 – association is observed only in one trait; PP.H3 – two traits are regulated by distinct underlying causal variants; PP.H4 – the traits are regulated by a shared underlying causal variant (colocalisation).

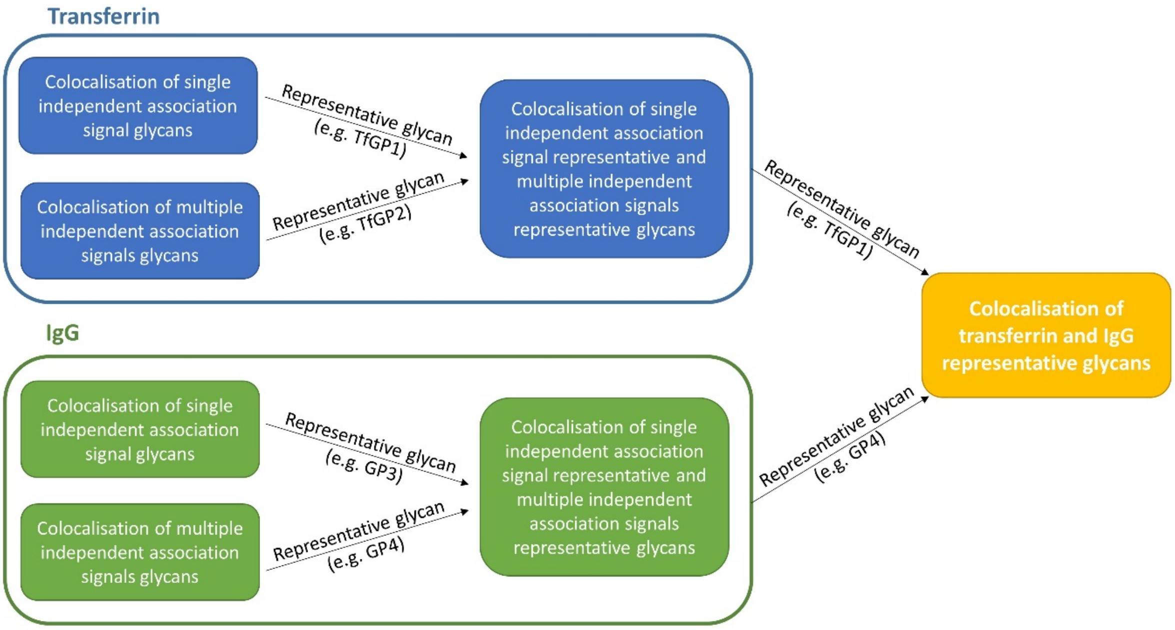

Supplementary Figure 6. Workflow applied for transferrin and IgG glycan traits colocalisation analysis. For each protein (i.e. transferrin and IgG) and each genomic region (i.e. *FUT8* and *FUT6*), the group of glycan traits showing multiple independent signals of association and, separately, the group of glycans carrying only one independent association signal at locus were pair-tested for colocalisation. Pairs of glycan traits with an ABF posterior probability for hypothesis 4 (suggestive of colocalisation) > 80% were pooled in the same colocalisation group. For each colocalisation group identified, the glycan trait reporting the lowest p-value was selected as group representative and carried on to the next step, where traits with single and multiple independent associations for each protein were tested for colocalisation. Similar to previous steps, glycan traits were grouped together on the basis of their colocalisation analysis results and the lowest p-value representative was chosen for the next step, where finally representative transferrin and IgG glycans at each locus were tested for colocalisation.

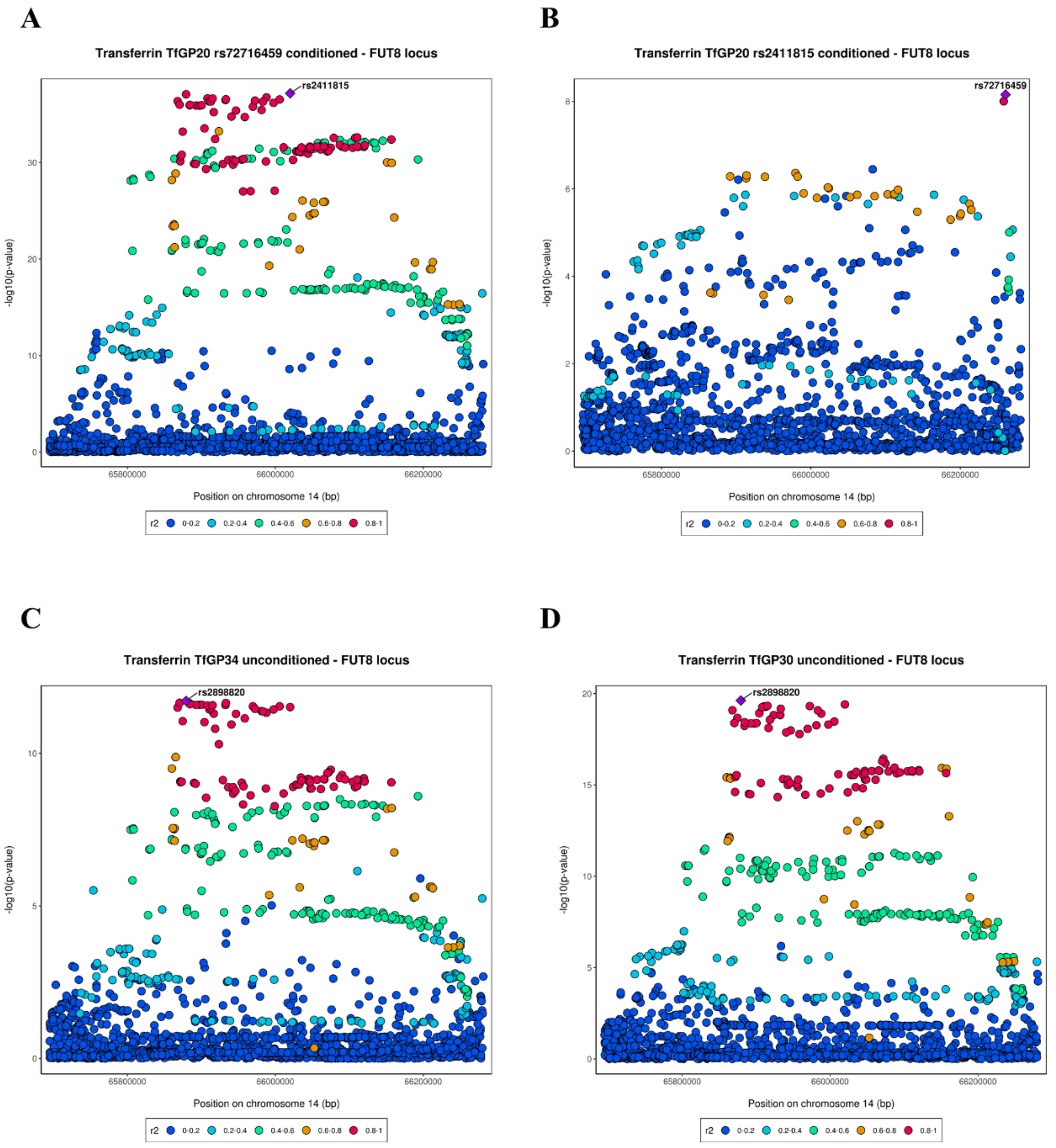

Supplementary Figure 7. Local association patterns of transferrin N-glycans tested for trait colocalisation at *FUT8* locus. Colocalisation analysis results for these glycan traits can be found at Supplementary Tables 15 (C and D) and 16 (A and B). Since TfGP20 also has an independent secondary association signal (see Supplementary Table 5), local association patterns are reported also for conditioned summary statistics.

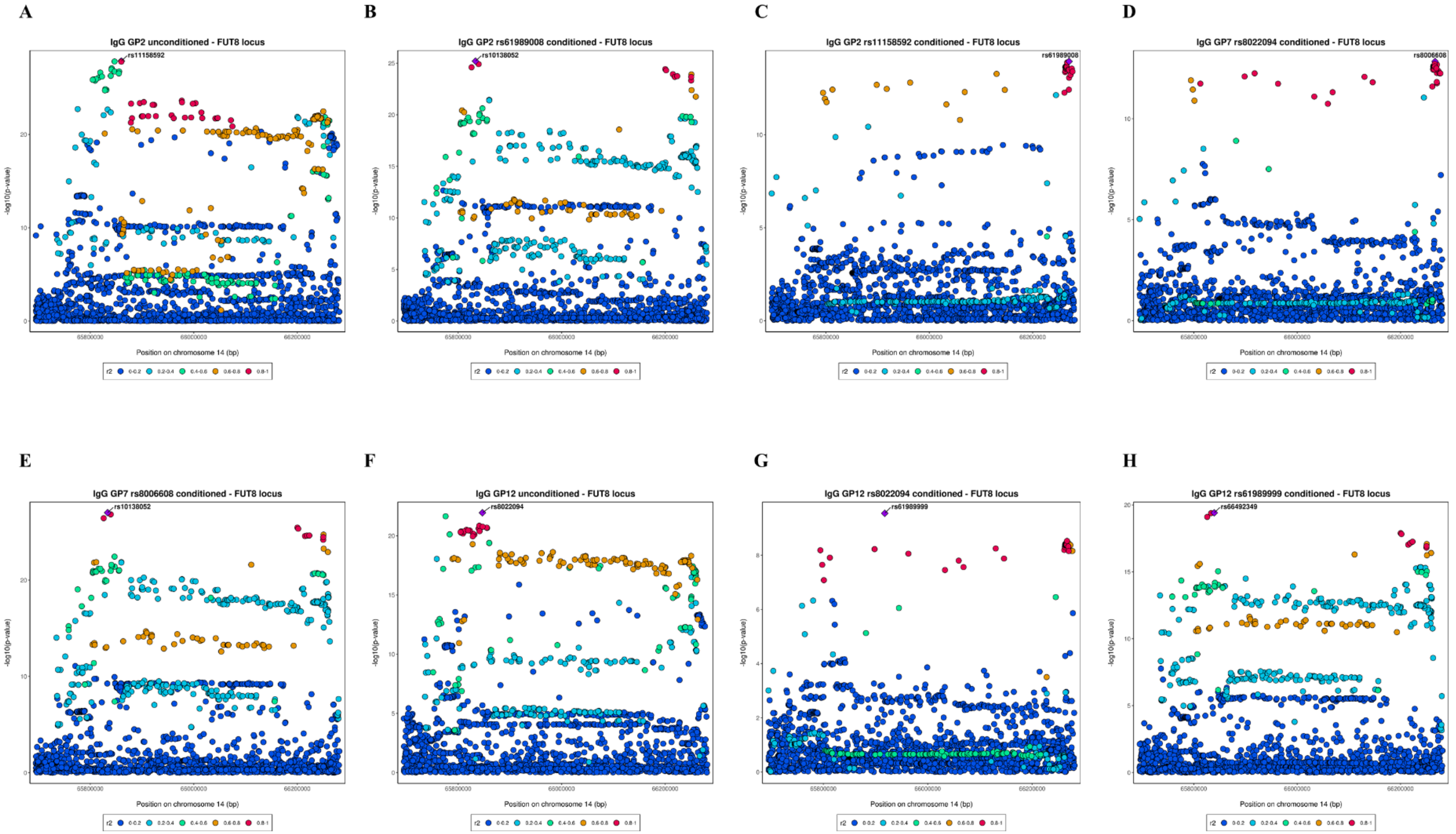
A
IgG GP2 unconditioned - FUT8 locus
rs11158592
B
IgG GP2 rs61989008 conditioned - FUT8 locus
rs10138052
C
IgG GP2 rs11158592 conditioned - FUT8 locus
rs61989008
D
IgG GP7 rs8022094 conditioned - FUT8 locus
rs8006608
E
IgG GP7 rs8006608 conditioned - FUT8 locus
rs10138052
F
IgG GP12 unconditioned - FUT8 locus
rs8022094
G
IgG GP12 rs8022094 conditioned - FUT8 locus
rs61989999
H
IgG GP12 rs61989999 conditioned - FUT8 locus
rs66492349
-log10(p-value)
Position on chromosome 14 (bp)
r2 0-0.2 0.2-0.4 0.4-0.6 0.6-0.8 0.8-1

I

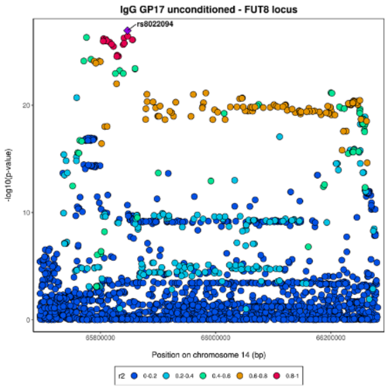

J

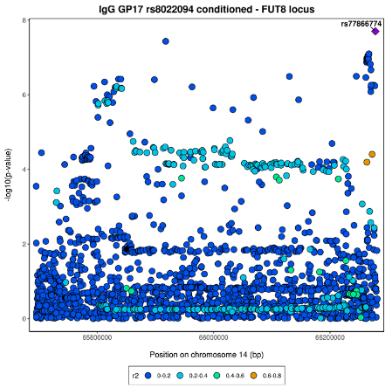

K

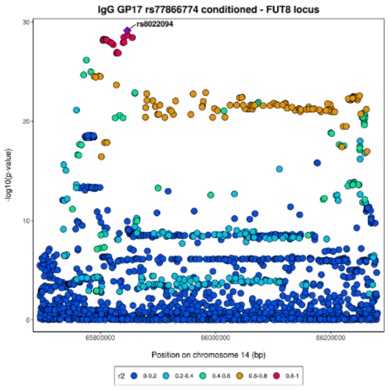

L

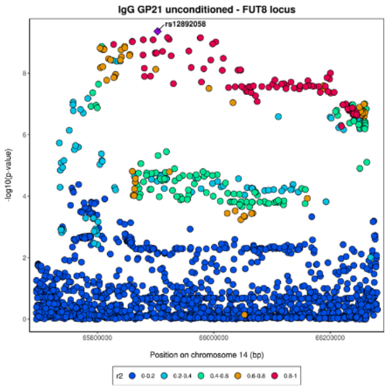

M

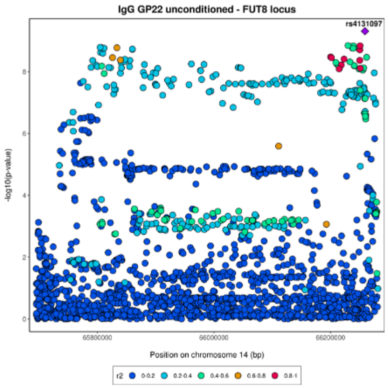

Supplementary Figure 8. Local association patterns of IgG N-glycans tested for trait colocalisation at *FUT8* locus. Colocalisation analysis results for these glycan traits can be found at Supplementary Table 15 (A, B, C, F, G, H, I, J, K, L, M and N) and 16 (D and E). Since GP7 also has an independent secondary association signal (see Supplementary Table 5), local association patterns are reported also for conditioned summary statistics.

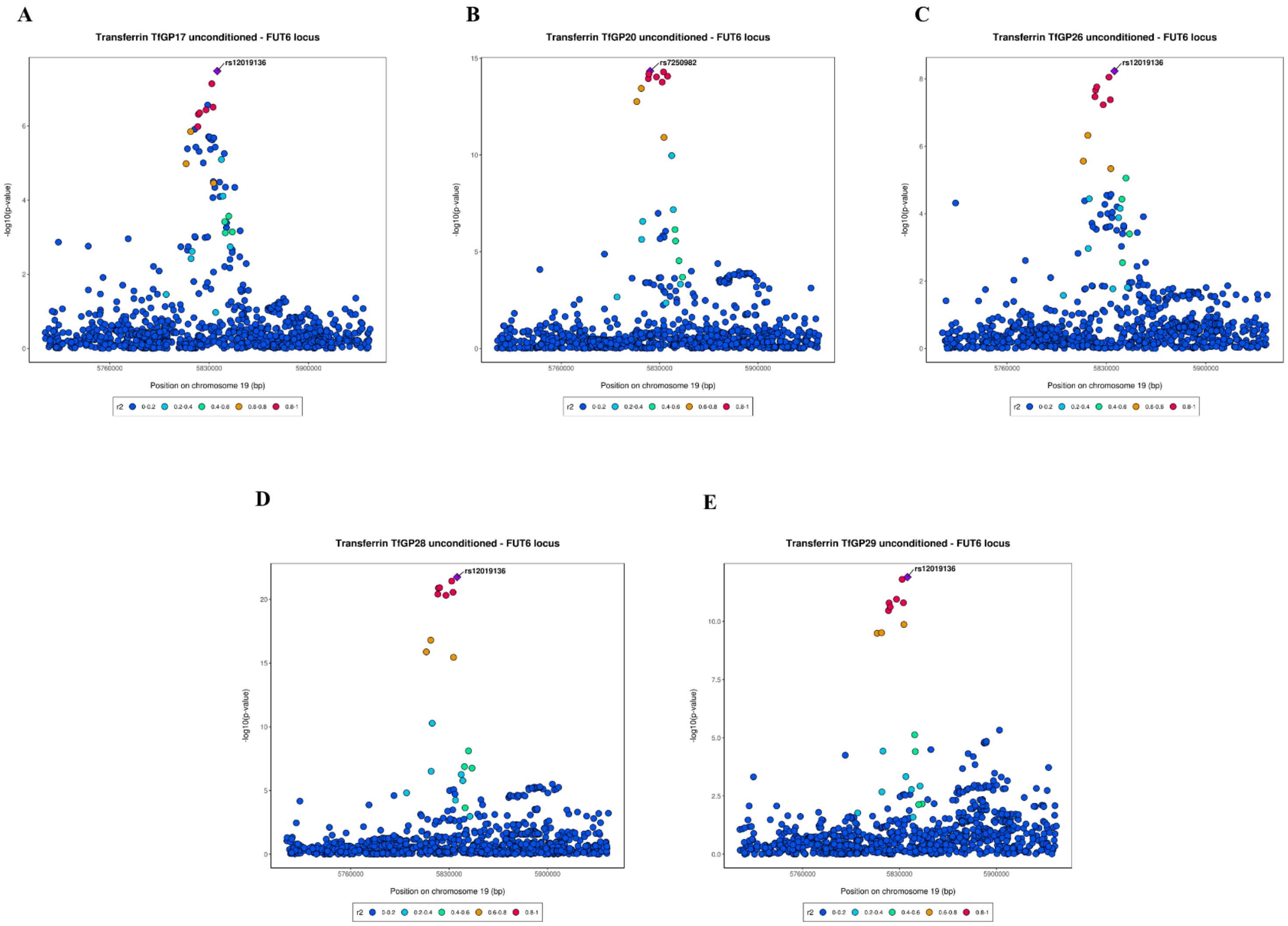

Supplementary Figure 9. Local association patterns of transferrin N-glycans tested for trait colocalisation at *FUT6* locus. Colocalisation analysis results for these glycan traits can be found at Supplementary Tables 15.

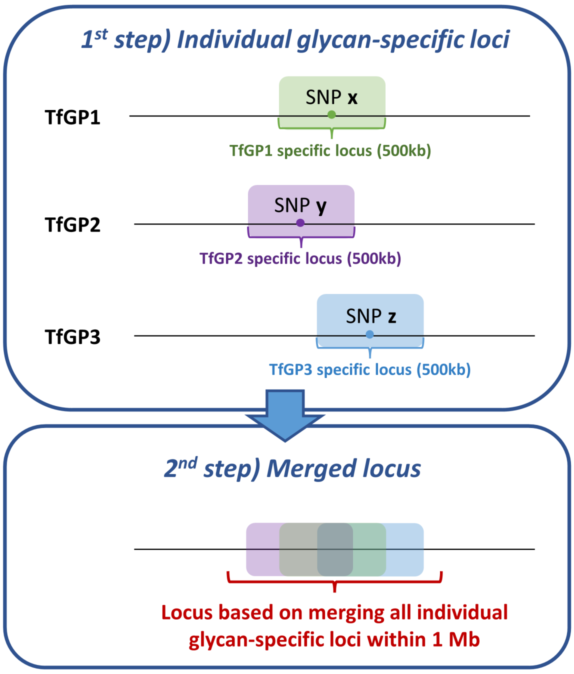

Supplementary Figure 10. Example of the workflow applied for loci definition. To define genomic regions significantly associated with N-glycan traits, we first grouped, for each glycan trait, all genetic variants located within a 500 kb window of the sentinel SNP (+/- 250 kb). With this 1st step we identified loci specific for each glycan trait. Then, to obtain a list of unique loci that are independent of the specific glycan trait, we grouped into a single locus all the glycan-sentinel SNP pairs that were overlapping within a 1Mb range. With this 2nd step we obtained a unique list of top glycan-sentinel SNP pairs, summarising the genomic regions most strongly associated with transferrin N-glycome across all traits.

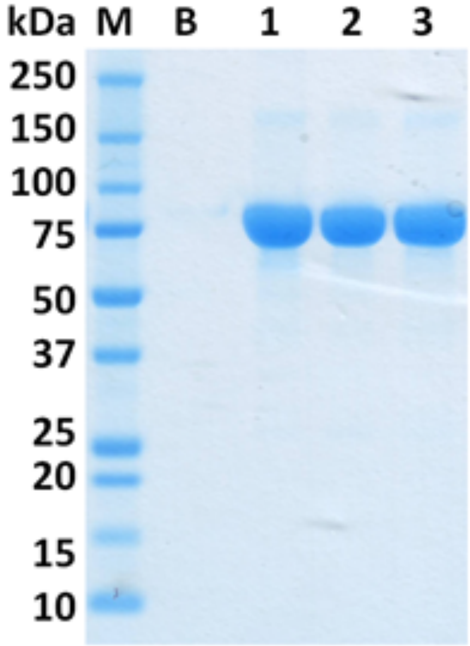

Supplementary Figure 11. Elution fractions (7.5× concentrated) after transferrin isolation from IgG depleted plasma (lanes 1-3) analysed by SDS-PAGE using 4–12 % Bis-Tris gradient gels (1.0 mm thickness) under reducing conditions according to the manufacturer's instructions (Life Technologies). The gels were run at 200 V for 35 min using a MES SDS buffering system. Protein bands were visualized by GelCode Blue staining reagent. M – Precision Plus Protein Standards All Blue molecular mass standard (Bio-Rad). B – blank sample (10 × concentrated). The same experiment was repeated 32 times (once for each plate) with similar results.

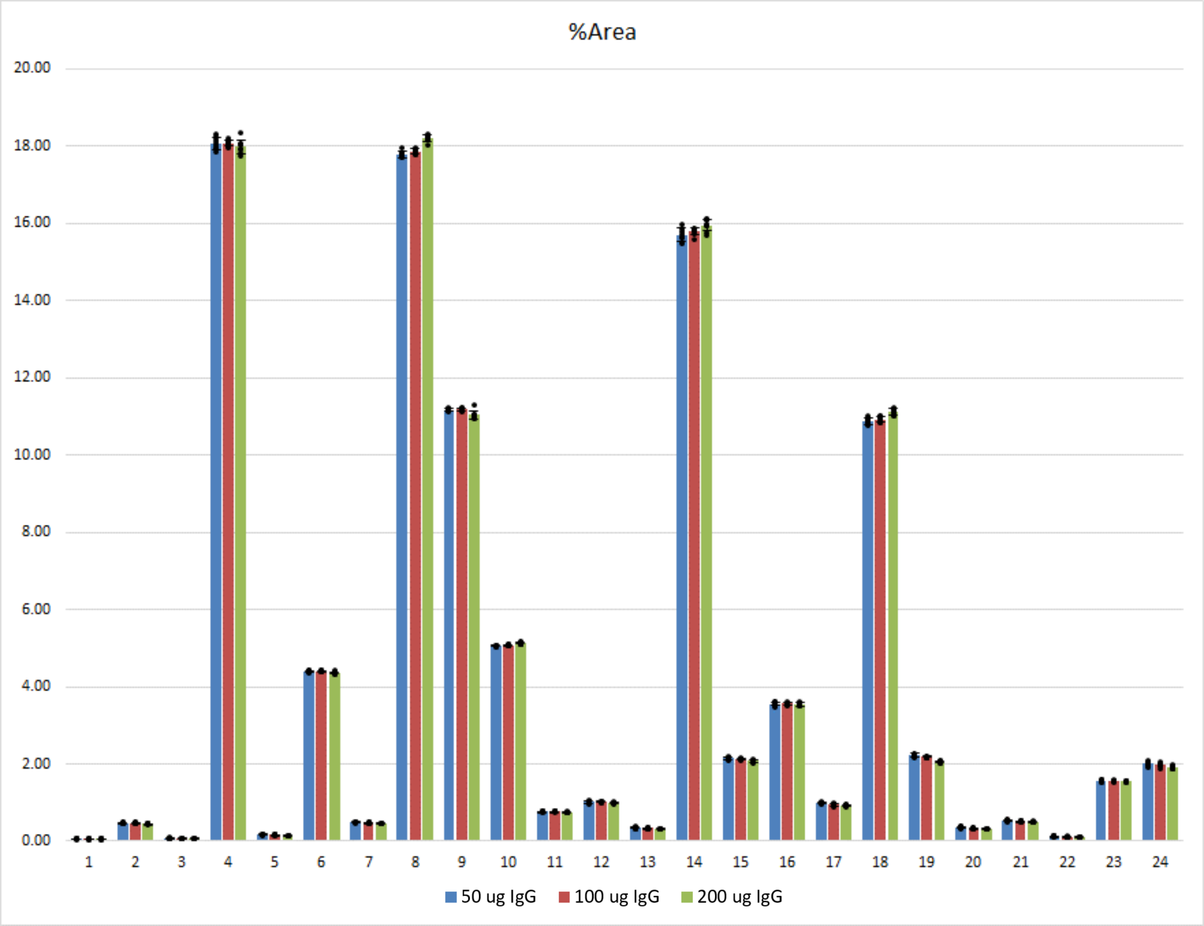

Supplementary Figure 12. Levels of IgG glycan traits (GP1-24) measured for different initial amounts of isolated IgG protein. Data are presented as mean +/- standard deviation, n=8 technical replicates for each amount of IgG. Corresponding data points are overlayed as dots.

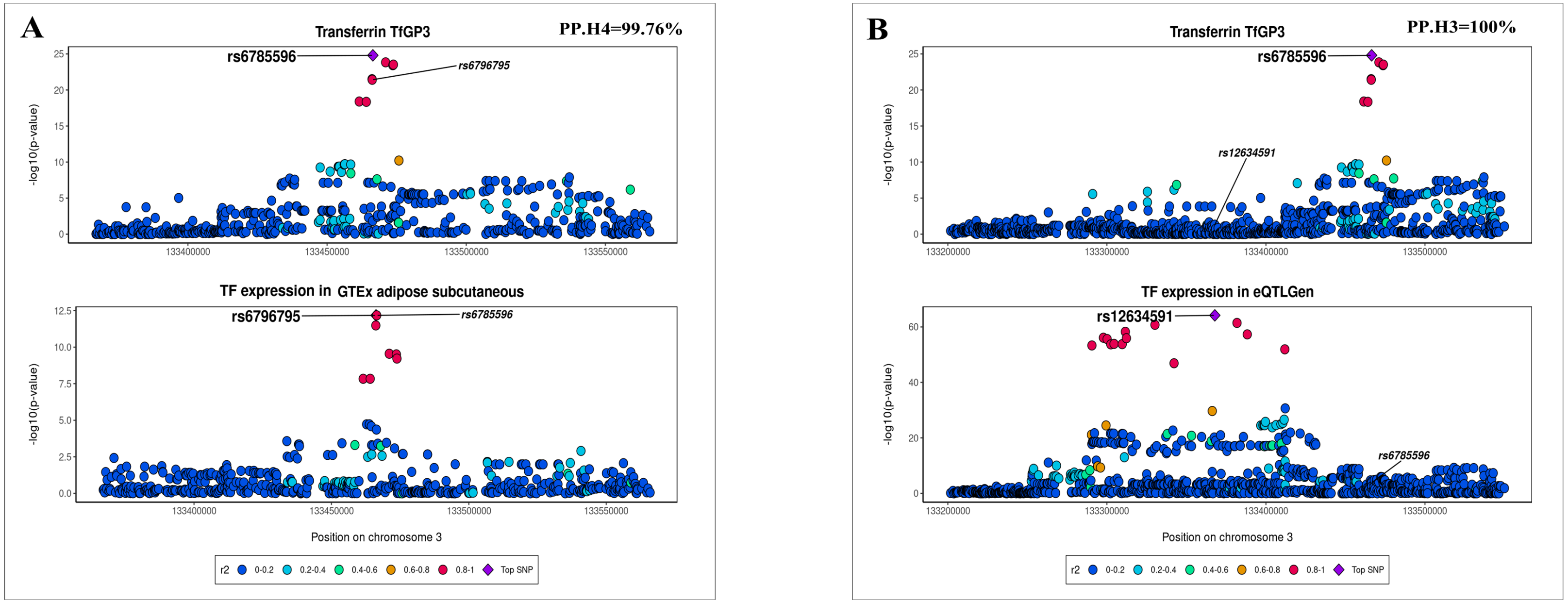

Supplementary Figure 13. Local association patterns of transferrin N-glycan TfGP3 and adipose tissue eQTLs (A) and blood (B) at TF gene locus. Adipose subcutaneous eQTLs were taken from GTEx v7[14], and gene expression data blood eQTLs were taken from eQTLGen consortium[15]. For each pairwise colocalisation test, the hypothesis having the higher posterior probability is reported at the top right of the plot. PP.H3 – two traits are regulated by distinct underlying causal variants; PP.H4 – the traits are regulated by a shared underlying causal variant (colocalisation).

Supplementary References

1. Trbojević-Akmačić, I. *et al.* Chromatographic monoliths for high-throughput immunoaffinity isolation of transferrin from human plasma. *Croat. Chem. Acta* **89**, 203–211 (2016).
2. Tyanova, S., Temu, T. & Cox, J. The MaxQuant computational platform for mass spectrometry-based shotgun proteomics. *Nat. Protoc.* **11**, 2301–2319 (2016).
3. Zheng, J. *et al.* Phenome-wide Mendelian randomization mapping the influence of the plasma proteome on complex diseases. *Nat. Genet.* **52**, 1122–1131 (2020).
4. Giambartolomei, C. *et al.* Bayesian Test for Colocalisation between Pairs of Genetic Association Studies Using Summary Statistics. *PLoS Genet.* **10**, (2014).
5. Klarić, L. *et al.* Glycosylation of immunoglobulin G is regulated by a large network of genes pleiotropic with inflammatory diseases. *Sci. Adv.* **6**, eaax0301 (2020).
6. Sharapov, S. Z. *et al.* Defining the genetic control of human blood plasma N-glycome using genome-wide association study. *Hum. Mol. Genet.* **28**, 2062–2077 (2019).
7. Huffman, J. E. *et al.* Polymorphisms in B3GAT1, SLC9A9 and MGAT5 are associated with variation within the human plasma N-glycome of 3533 European adults. *Hum. Mol. Genet.* **20**, 5000–5011 (2011).
8. Bermingham, M. L. *et al.* N-glycan profile and kidney disease in type 1 diabetes. *Diabetes Care* **41**, 79–87 (2018).
9. Benyamin, B. *et al.* Novel loci affecting iron homeostasis and their effects in individuals at risk for hemochromatosis. *Nat. Commun.* **5**, 4926 (2014).
10. Ziyatdinov, A. *et al.* lme4qtl: Linear mixed models with flexible covariance structure for genetic studies of related individuals. *BMC Bioinformatics* **19**, (2018).
11. Karssen, L. C., van Duijn, C. M. & Aulchenko, Y. S. The GenABEL Project for statistical genomics. *F1000Research* **5**, (2016).
12. Bycroft, C. *et al.* The UK Biobank resource with deep phenotyping and genomic data. *Nature* **562**, 203–209 (2018).
13. Kutalik, Z. *et al.* Genome-wide association study identifies two loci strongly affecting transferrin glycosylation. *Hum. Mol. Genet.* **20**, 3710–7 (2011).
14. Lonsdale, J. *et al.* The Genotype-Tissue Expression (GTEx) project. *Nature Genetics* **45**, 580–585 (2013).
15. Võsa, U. *et al.* Large-scale cis- and trans-eQTL analyses identify thousands of genetic loci and polygenic scores that regulate blood gene expression. *Nat. Genet. 2021 539* **53**, 1300–1310 (2021).
