# Supplementary material for: Genetic regulation of post-translational modification of two distinct proteins
Source: Nat Commun. 2022 Mar 24;13:1586. doi: 10.1038/s41467-022-29189-5 (PMC8948205; doi:10.1038/s41467-022-29189-5)
Supplement: Supplementary file 3 — Description of Additional Supplementary Files [file 41467_2022_29189_MOESM3_ESM.pdf]

Description of Additional Supplementary Files

Supplementary Data 1. Loci genome-wide significantly associated with at least one of the transferrin 35 N-glycan traits in CROATIA-Korcula cohort. Each locus is represented by the SNP with the strongest association in the region. The Bonferroni-corrected genome-wide significance threshold corresponds to 1.43×10-9. Glycosyltransferase loci are reported at the top of the Table, while other loci are listed at the bottom of the Table. Locus, coded by "chromosome: locus start–locus end" according to GRCh37 human genome build; Genes, suggested candidate genes; SNP, variant with the strongest association in the locus; EA, SNP allele for which effect estimate is reported; OA, SNP other allele; EAF, frequency of the effect allele; Glycan, glycan trait associated with the reported SNP; Phe. var., proportion of variance in phenotype explained by the strongest associated SNP; No. of glycans, number of other glycan traits significantly associated with variants in given locus; Beta, effect estimate for the SNP and glycan with the strongest association in the locus; SE, standard error of the effect estimate, P, p-value for the effect estimate; P replication, p-value for the effect estimate in replication cohort (VIKING) when using replication-discovery approach. P-values are derived from two-sided Wald test with one degree of freedom. P-values are derived from two-sided Wald test with one degree of freedom.

Supplementary Data 2. Loci genome-wide significantly associated with at least one of the transferrin 35 N-glycan traits in VIKING cohort. Each locus is represented by the SNP with the strongest association in the region. The Bonferroni-corrected genome-wide significance threshold corresponds to 1.43×10-9. Glycosyltransferase loci are reported at the top of the Table, while other loci are listed at the bottom of the Table. Locus, coded by "chromosome: locus start–locus end" according to GRCh37 human genome build; Genes, suggested candidate genes; SNP, variant with the strongest association in the locus; EA, SNP allele for which effect estimate is reported; OA, SNP other allele; EAF, frequency of the effect allele; Glycan, glycan trait associated with the reported SNP; Phe. var., proportion of variance in phenotype explained by the strongest associated SNP; No. of glycans, number of other glycan traits significantly associated with variants in given locus; Beta, effect estimate for the SNP and glycan with the strongest association in the locus; SE, standard error of the effect estimate, P, p-value for the effect estimate. P-values are derived from two-sided Wald test with one degree of freedom.

Supplementary Data 3. Heritability estimates for each transferrin glycan trait in CROATIA-Korcula and VIKING cohorts.

Supplementary Data 4. Summary of all transferrin N-glycan trait genome-wide significant associations. For each transferrin glycan trait, all genome-wide significantly associated loci are reported. The Bonferroni-corrected genome-wide significance threshold corresponds to 1.43×10-9. Glycan, glycan trait associated with the reported SNP; Locus, coded by "chromosome: locus start–locus end" according to GRCh37 human genome build; SNP, variant with the strongest association in the locus for the glycan trait; Chr, chromosome where the SNP is located; Pos, base pair position of the SNP on the chromosome; EA, SNP

allele for which effect estimate is reported; Beta, effect estimate for the SNP and glycan with the strongest association in the locus; SE, standard error of the effect estimate; P, p-value for the effect estimate (two sided Wald test with one degree of freedom); Candidate gene, suggested candidate gene associated with transferrin N-glycans in the present study; Gene description, function of the product coded by the gene.

Supplementary Data 5. Secondary association signals at transferrin and IgG glycan traits associated loci. Secondary association signals were identified by conditional and joint analysis on N-glycan traits GWAS summary statistics. The Bonferroni-corrected genome-wide significance threshold corresponds to 1.43×10-9 for transferrin, and to 2.08×10-9 for IgG glycan traits. Protein, protein of glycan traits (i.e. transferrin or IgG); Glycan, glycan trait associated with the reported SNP; Gene, suggested candidate gene; SNP, variant reporting a significant association with glycan trait at the locus; LD_r, linkaged disequilibrium R between the given and the following SNP; Chr, chromosome; Pos, SNP physical position; EA, SNP allele for which effect estimate is reported; EAF, frequency of the effect allele; Phe. var. - proportion of variance in phenotype explained by the associated SNP; Beta, effect estimate for the SNP and glycan association from the original meta-analysis; SE, standard error of the effect estimate from the original meta-analysis; P GC, p-value for the effect estimate from the original meta-analysis, adjusted by the genomic control method; BetaJ, effect estimate for the SNP and glycan association from a joint analysis of all the selected SNPs; BetaJ se, standard error of the effect estimate from a joint analysis of all the selected SNPs; PJ GC, p-value for the effect estimate from a joint analysis of all the selected SNPs, adjusted by the genomic control method.

Supplementary Data 6: Assessing the impact of transferrin pQTL (proxy for transferrin protein levels) on transferrin glycan levels. Association of pQTL with glycan levels was assessed using the likelihood ratio test between the following models:

M0: glycan ~ age + sex

M1: glycan ~ age + sex + pQTL (rs8177240)

M2: glycan ~ age + sex + glyQTL (rs6785596)

M3: glycan ~ age + sex + pQTL (rs8177240) + glyQTL (rs6785596)

P - p value of the likelihood ratio test between two models (df = 1), LogLik - log likelihood, Chisq - chi squared statistic of the likelihood ratio test. In bold statistically significant ($p<=0.05/35=1.4E-3$) likelihood ratio tests. Adj R2 - percentage of variance explained (coefficient of determination) by the given model (M), accounting for the number of covariates in the model, estimated by a linear fixed effect model on relatedness corrected glycan traits

Supplementary Data 7. Transferrin N-glycans associated genes reported by previous N-glycome GWAS. Locus, loci associated with transferrin N-glycans, coded by “chromosome: locus start–locus end” according to GRCh37 human genome build; Gene, suggested

candidate gene associated with transferrin N-glycans in the present study and also reported by previous N-glycome GWAS; Coded product, function of the product coded by the gene; Glycan, transferrin N-glycan trait showing the strongest association for that genomic region; IgG glycan trait, description of the IgG glycan trait showing the strongest association with the genomic region as reported in (Klarić et al., 2020); Total plasma glycan trait, description of the total plasma proteins glycan trait showing the strongest association with the genomic region as reported in (Sharapov et al., 2019); Publication(s), previous N-glycome association study or studies reporting the candidate gene.

Supplementary Data 8a. Phenoscanner results for overlap of transferrin N-glycans associated SNPs (and their proxies) with gene expression (eQTL). snp, the input rsID; ref_hg19_coordinates, hg19 chromosome position for ref_rsid; ref_hg38_coordinates, hg38 chromosome position for ref_rsid; ref_a1, the effect allele (aligned to the + strand) for ref_rsid; ref_a2, the non-effect allele (aligned to the + strand) for ref_rsid; rsid, rsID for which PhenoScanner results are reported; hg19_coordinates, the hg19 chromosome position for rsid; hg38_coordinates, the hg38 chromosome position for rsid; a1, the effect allele (aligned to the + strand) for rsid; a2, the non-effect allele (aligned to the + strand) for rsid; proxy, an indicator variable which equals 0 if the proxy SNP is the input SNP and 1 otherwise; r2, the r2 between the input SNP and the proxy SNP based on the phased haplotypes from 1000 Genomes; dprime, the D' between the input SNP and the proxy SNP based on the phased haplotypes from 1000 Genomes; trait, phenotype or disease; efo, the EFO ontology term for the phenotype or disease; study, the name of the consortium/lead author of the study; pmid, PubMed ID; ancestry, the ancestry of the study; year, the year the study was published; tissue, the tissue in which the gene expression was measured (eQTL dataset only); exp_gene, the HGNC ID for the expressed gene (eQTL dataset only); exp_ensembl, the Ensembl ID for the expressed gene (eQTL dataset only); probe, the probe for the expressed gene (eQTL dataset only); beta, association between the trait and the SNP expressed per additional copy of the effect allele (odds ratios are given on the log-scale); se, standard error of beta; p, p-value (for information regarding the statistical test used to derive the reported p-value, refer to the publication reported in the pmid column); direction, the direction of association with respect to the effect allele; n, number of individuals; n_studies, number of studies; unit, unit of analysis (IVNT stands for inverse normally rank transformed phenotype); dataset, the dataset ID.

Supplementary Data 8b. SMR-HEIDI results for colocalisation of transferrin N-glycans associated SNPs with gene expression (eQTL). Coloured cells depict significant SMR coefficients, with P HEIDI > 0.001 in green and P HEIDI < 0.001 in yellow. GWAS1, transferrin glycan trait; GWAS2, gene expression trait; Locus, SNP used as instrumental variable; Gene GWAS1, candidate gene prioritised in GWAS1 (the present study); #SNPs HEIDI, number of SNPs used in the HEIDI test; P HEIDI, p-value for heterogeneity test; P SMR, nominal, two-sided p-value for the MR effect estimate; Beta SMR, MR effect estimate; Gene GWAS2, candidate gene prioritised in GWAS2; P GWAS2, -log10 of the nominal, two-sided p-value of the SNP association with GWAS2; Probe GWAS2, probe ID;

Collection, resource from which summary statistics for gene expression levels and complex traits were obtained; Tissue, the tissue for which gene expression data is reported.

Supplementary Data 9. Results of VEP analysis for transferrin N-glycome strongest associated SNPs (and their proxies). Uploaded variation, as chromosome_start_alleles; Location, in standard coordinate format (chr:start or chr:start-end); Allele, the variant allele used to calculate the consequence; Consequence, consequence type of this variant; Gene, name of affected gene; Feature type, type of feature (i.e. Transcript, RegulatoryFeature, MotifFeature); Feature, Ensembl sTable ID of feature; BIOTYPE, Biotype of transcript or regulatory feature; cDNA position, relative position of base pair in cDNA sequence; CDS position, relative position of base pair in coding sequence; Protein position, relative position of amino acid in protein; Amino acid change, only given if the variant affects the protein-coding sequence; Codon change, the alternative codons with the variant base in upper case; Existing variation- known identifier of existing variant; SIFT, the SIFT prediction and/or score, with both given as prediction(score); PolyPhen, the PolyPhen prediction and/or score; AF, Frequency of existing variant in 1000 Genomes; PUBMED, Pubmed ID(s) of publications that cite existing variant.

Supplementary Data 10. Results of RSAT matrix-scan analysis for transcription factor motif alterations by transferrin N-glycosylation associated SNPs. Multiple motifs, spanning across the same sequence, were identified as binding sites of HNF1a transcription factor at FUT8 locus. Only the statistically most likely motif is reported in the Table. Chr, chromosome; SNP, strongest associated variant in the locus and centre of the sequence tested as transcription factor binding site; Gene, candidate gene prioritised in the present study; Start pos, starting position of the sequence tested as transcription factor binding site; End pos, ending position of the sequence tested as transcription factor binding site; Transcription factor, transcription factor tested for binding at the input sequence; PWM start, position in the input sequence where the position weight matrix (PWM) starts; PWM end, position in the input sequence where the PWM ends; PWM sequence, sequence of the PWM; PWM weight, weight score of PWM match, calculated by the log ratio of the probability of the matrix sequence given the input sequence and the probability of the matrix sequence given the background model; P, the probability of observing the weight at least as good when the motif is compared with random sequences (background model). With a p-value of 0.00001 a false positive prediction is expected every 100 kilobases (Turatsinze et al, 2008)

Supplementary Data 11a. Phenoscanner results for overlap of transferrin N-glycans associated SNPs (and their proxies) with complex traits and diseases. snp, the input rsID; ref_hg19_coordinates, hg19 chromosome position for ref_rsid; ref_hg38_coordinates, hg38 chromosome position for ref_rsid; ref_a1, the effect allele (aligned to the + strand) for ref_rsid; ref_a2, the non-effect allele (aligned to the + strand) for ref_rsid; rsid, rsID for which PhenoScanner results are reported; hg19_coordinates, the hg19 chromosome position for rsid; hg38_coordinates, the hg38 chromosome position for rsid; a1, the effect allele (aligned to the + strand) for rsid; a2, the non-effect allele (aligned to the + strand) for rsid; proxy, an indicator variable which equals 0 if the proxy SNP is the input SNP and 1

otherwise; r2, the r2 between the input SNP and the proxy SNP based on the phased haplotypes from 1000 Genomes; dprime, the D' between the input SNP and the proxy SNP based on the phased haplotypes from 1000 Genomes; trait, phenotype or disease; efo, the EFO ontology term for the phenotype or disease; study, the name of the consortium/lead author of the study; pmid, PubMed ID; ancestry, the ancestry of the study; year, the year the study was published; beta, association between the trait and the SNP expressed per additional copy of the effect allele (odds ratios are given on the log-scale); se, standard error of beta; p, p-value (for information regarding the statistical test used to derive the reported p-value, refer to the relative publication highlighted in the pmid column); direction, the direction of association with respect to the effect allele; n, number of individuals; n_cases, number of cases; n_controls, number of controls; n_studies, number of studies; unit, unit of analysis (IVNT stands for inverse normally rank transformed phenotype); dataset, the dataset ID.

Supplementary Data 11b. SMR-HEIDI results for colocalisation of transferrin N-glycans associated SNPs with complex traits and diseases. Coloured cells depict significant SMR coefficients with P HEIDI > 0.001. GWAS1, transferrin glycan trait; GWAS2, complex trait or disease; Locus, SNP used as instrumental variable; Gene GWAS1, candidate gene prioritised in GWAS1 (the present study); #SNPs HEIDI, number of SNPs used in the HEIDI test; P HEIDI, p-value for heterogeneity test; P SMR, nominal, two-sided p-value for the MR effect estimate; Beta SMR, MR effect estimate; P GWAS2, -log10 of the nominal, two-sided p-value of the SNP association with GWAS2.

Supplementary Data 12. Results of transferrin N-glycome and complex human traits and diseases bi-directional two-sample Mendelian Randomisation. Exposure, (glycan or complex/disease) trait tested as exposure; Outcome, (glycan or complex/disease) trait tested as outcome; MR method, method used to calculate the MR effect: Wald ratio for single instrument SNPs, inverse-variance-weighted test for multiple instruments; N, sample size of the outcome GWAS summary statistics; No. of SNPs, number of instrumental SNPs; Beta, MR effect estimate; SE - standard error of the MR effect estimate; P - nominal, two-sided p-value of the MR effect estimate.

Supplementary Data 13. Results of transferrin N-glycome and complex human traits and diseases colocalisation analysis. Transferrin glycan, transferrin glycan trait tested; Complex trait, complex human trait or disease tested; Gene, suggested candidate gene for transferrin glycosylation; No. of SNPs, number of SNPs included in the colocalisation test; PP.H1, posterior probability percentage for hypothesis 1 (i.e. association at the locus only for trait 1); PP.H2, posterior probability percentage for hypothesis 2 (i.e. association at the locus only for trait 2); PP.H3, posterior probability percentage for hypothesis 3 (i.e. association of traits at the same locus but due to two independent causal variants); PP.H4, posterior probability percentage for hypothesis 4 (i.e. traits colocalisation, where association of traits at the same locus is due to a single causal variant).

Supplementary Data 14. Loci genome-wide significantly associated with at least one of the 24 IgG N-glycan traits in GWAMA. Each locus is represented by the SNP reporting the strongest association in the region. Locus, coded by “chromosome: locus start–locus end”

according to GRCh37 human genome build; Genes, suggested candidate genes as reported by (Klarić et al., 2020). For loci not listed in (Klarić et al., 2020), no candidate gene was reported; SNP, variant with the strongest association in the locus; EA, SNP allele for which effect estimate is reported; OA, SNP other allele; EAF, frequency of the effect allele; No. of SNPs, number of SNPs independently contributing to trait variation according to GCTA-COJO; Glycan, glycan trait associated with the reported SNP (IGP - nomenclature used in Klaric et al); Phe. var., proportion of variance in phenotype explained by the strongest associated SNP; No. of glycans, number of other glycan traits significantly associated with variants in given locus; Beta, effect estimate for the SNP and glycan with the strongest association in the locus; SE, standard error of the effect estimate, P, p-value for the effect estimate (two-sided Wald test with one degree of freedom). The Bonferroni-corrected genome-wide significance threshold corresponds to 2.08×10-9

Supplementary Data 15. Results of transferrin and IgG N-glycome colocalisation analysis within single protein at FUT8 and FUT6 loci. Step, step in the colocalisation analysis procedure (i.e. step testing glycan traits showing multiple independent signals of association at locus, step testing glycan traits carrying only one independent association signal at locus, step testing the representatives for glycan traits with multiple independent signals of association and for glycan traits with a single independent signal of association) (see Supplementary Figure 6); Protein, protein of glycan traits (i.e. transferrin or IgG); Gene, suggested candidate gene; Glycan 1, first element of the couple of glycan traits tested; Glycan 1 P, meta-analysis p-value of Glycan 1; Glycan 2, second element of the couple of glycan traits tested; Glycan 2 P, meta-analysis p-value of Glycan 2; PP.H3, posterior probability percentage for hypothesis 3 (i.e. association of traits at the same locus but due to two independent causal variants); PP.H4, posterior probability percentage for hypothesis 4 (i.e. trait colocalisation, where association of traits at the same locus is due to a single causal variant); Rep glycan, glycan trait chosen as colocalisation group representative (i.e. within a group of glycan traits colocalising at the locus, the glycan trait reporting the lowest p-value).

Supplementary Data 16. Results of transferrin and IgG N-glycome colocalisation analysis between proteins at FUT8 and FUT6 loci. Gene, suggested candidate gene; Transferrin glycan, transferrin glycan trait of the couple of glycan traits tested; Transferrin glycan P, meta-analysis p-value of Transferrin glycan; IgG glycan, IgG glycan trait of the couple of glycan traits tested; IgG glycan P, p-value of IgG glycan; PP.H3, posterior probability percentage for hypothesis 3 (i.e. association of traits at the same locus but due to two independent causal variants); PP.H4, posterior probability percentage for hypothesis 4 (i.e. traits colocalisation, where association of traits at the same locus is due to a single causal variant).

Supplementary Data 17. CROATIA-Korcula and VIKING cohort sample demographics, details of genotyping and imputation. ID call rate, percentage of SNPs successfully genotyped in every individual; MAF, minor allele frequency; SNP call rate, percentage of samples in which the given genotype was successfully called; HWE P, Hardy-Weinberg Equilibrium p-value.

Supplementary Data 18. Full list of tissue and complex traits GWAS used in SMR-HEIDI analysis, MR and colocalisation analysis. Collection, resource from which summary statistics of gene expression levels and complex traits were obtained for SMR-HEIDI analysis; Trait abbreviation, tissue or complex trait abbreviation; Trait full name, tissue or complex trait full name; PMID or link to GWAS, study from which summary statistics of gene expression levels and complex traits were obtained for SMR-HEIDI analysis; Comment, remark regarding the summary statistics of gene expression levels and complex traits used for SMR-HEIDI analysis; PMID or link to replication GWAS, study from which summary statistics of complex traits were obtained for MR and colocalisation analysis.

Supplementary Data 19. Transferrin TfGP3 and TfGP8 glycans association signals at chromosome 3 before and after conditioning by transferrin pQTL. SNP, variant with the strongest association in the locus for the glycan trait; SNP_id, SNP location coded as "chromosome:base pair position" according to GRCh37 human genome build; EA - SNP allele for which the effect estimate is reported; EAF - frequency of the effect allele; Glycan, glycan trait associated with the reported SNP; Beta, effect estimate for the SNP associated with the glycan; Beta C, effect estimate for the SNP associated with the glycan conditioned by transferrin pQTL; SE, standard error of the effect estimate; SE C, standard error of the effect estimate conditioned by transferrin pQTL; P, p-value for the effect estimate ; P C, p-value for the effect estimate conditioned by transferrin pQTL. P-values were derived from the two-sided Wald test with one degree of freedom
