# Supplementary material for: Genetic regulation of post-translational modification of two distinct proteins
Source: Nat Commun. 2022 Mar 24;13:1586. doi: 10.1038/s41467-022-29189-5 (PMC8948205; doi:10.1038/s41467-022-29189-5)
Supplement: Supplementary file 5 — Reporting Summary [file 41467_2022_29189_MOESM5_ESM.pdf]

nature portfolio

Corresponding author(s): Lucija Klaric

Last updated by author(s): Jan 28, 2022

Reporting Summary

Nature Portfolio wishes to improve the reproducibility of the work that we publish. This form provides structure for consistency and transparency in reporting. For further information on Nature Portfolio policies, see our Editorial Policies and the Editorial Policy Checklist.

Statistics

For all statistical analyses, confirm that the following items are present in the figure legend, table legend, main text, or Methods section.

n/a	Confirmed	
☐	☒	The exact sample size (*n*) for each experimental group/condition, given as a discrete number and unit of measurement
☐	☒	A statement on whether measurements were taken from distinct samples or whether the same sample was measured repeatedly
☐	☒	The statistical test(s) used AND whether they are one- or two-sided<br>*Only common tests should be described solely by name; describe more complex techniques in the Methods section.*
☐	☒	A description of all covariates tested
☐	☒	A description of any assumptions or corrections, such as tests of normality and adjustment for multiple comparisons
☐	☒	A full description of the statistical parameters including central tendency (e.g. means) or other basic estimates (e.g. regression coefficient) AND variation (e.g. standard deviation) or associated estimates of uncertainty (e.g. confidence intervals)
☐	☒	For null hypothesis testing, the test statistic (e.g. *F*, *t*, *r*) with confidence intervals, effect sizes, degrees of freedom and *P* value noted<br>*Give P values as exact values whenever suitable.*
☐	☒	For Bayesian analysis, information on the choice of priors and Markov chain Monte Carlo settings
☒	☐	For hierarchical and complex designs, identification of the appropriate level for tests and full reporting of outcomes
☒	☐	Estimates of effect sizes (e.g. Cohen's *d*, Pearson's *r*), indicating how they were calculated

Our web collection on statistics for biologists contains articles on many of the points above.

Software and code

Policy information about availability of computer code

Data collection	MaxQuant software and the Empower 3 software, build 3471 (Waters) were used for glycan traits measurements, as detailed in the Supplementary Material and methods. Genetic data quality control and imputation was performed as detailed in Supplementary Table 17 and in referenced literature (PMID: 19260141 for CROATIA-Korcula and PMID: 18760389 for VIKING cohort).
Data analysis	Normalisation and batch correction of glycan traits was performed with sva 3.34.0 R package, rank transformation and covariate adjustment of glycan traits with GenABEL 1.1-6 R package. Tranferrin and IgG single cohort GWAS was performed with RegScan v. 0.5. IgG and transferrin meta-analysis was performed using METAL version 2011-03-25. Impact of transferrin protein levels on transferrin glycome associations was assessed with lmtest 0.9-38 and lme4qtl 0.2.2 R packages. Conditional analysis was performed with GCTA 1.91.4beta, LD-reference data handling was performed using Plink 2.0. Gene prioritisation was performed with FUMA v1.3.5e, VEP v 97. and RSAT v2018-08-04. PhenoScanner v1.1 database was used to "phenome scan" significant transferrin glycosylation SNPs. SMR-HEIDI was performed using the in-house implementation of the algorithm. Mendelian randomization analyses were performed using the TwoSampleMR 0.5.6 R package. Colocalisation analyses were performed using the coloc 4.0-6 R package. Local association plots were created using locuszoom v1.4. Analyses were performed using R 3.6.0., 3.6.1 and 4.0.2.

For manuscripts utilizing custom algorithms or software that are central to the research but not yet described in published literature, software must be made available to editors and reviewers. We strongly encourage code deposition in a community repository (e.g. GitHub). See the Nature Portfolio guidelines for submitting code & software for further information.

Data

Policy information about availability of data

All manuscripts must include a data availability statement. This statement should provide the following information, where applicable:

- Accession codes, unique identifiers, or web links for publicly available datasets
- A description of any restrictions on data availability
- For clinical datasets or third party data, please ensure that the statement adheres to our policy

The full summary statistics from the GWAS of 35 transferrin glycan traits and 24 IgG glycan traits generated in this study have been deposited in the DataShare repository [https://datashare.ed.ac.uk/handle/10283/4088]. There is neither Research Ethics Committee approval, nor consent from individual participants, to permit open release of the individual level research data underlying this study. The datasets analysed during the current study are therefore not publicly available. Instead, the research data and/or DNA samples are available from accessQTL@ed.ac.uk on reasonable request, following approval by the QTL Data Access Committee and in line with the consent given by participants. Each approved project is subject to a data or materials transfer agreement (D/MTA) or commercial contract. The UK Biobank genotypic data used in this study were approved under application 19655 and are available to qualified researches via the UK Biobank data access process [http://www.ukbiobank.ac.uk/register-apply/]. The position-specific scoring matrices (PSSMs) for HNF1A and FOXI1 genes used in this study are available in the JASPAR66 database under the accession code MA0046.2 [http://jaspar.genereg.net/api/v1/matrix/MA0046.2/?format=transfac] and MA0042.1 [http://jaspar.genereg.net/api/v1/matrix/MA0042.1/?format=transfac], respectively. The summary statistics for gene expression levels in tissues/cell types used in this study are available in the Blood eQTL study [http://cnsgenomics.com/software/smr/#eQTLsummarydata], in the CEDAR project [http://cedar-web.giga.ulg.ac.be/], in the GTEx project version 7 [https://gtexportal.org] and in the eQTLGen consortium [https://www.eqtlgen.org/]. The summary statistics for complex traits are available in various publicly available resources, as detailed in Supplementary Table 18.

Field-specific reporting

Please select the one below that is the best fit for your research. If you are not sure, read the appropriate sections before making your selection.

☒ Life sciences ☐ Behavioural & social sciences ☐ Ecological, evolutionary & environmental sciences

For a reference copy of the document with all sections, see nature.com/documents/nr-reporting-summary-flat.pdf

Life sciences study design

All studies must disclose on these points even when the disclosure is negative.

Sample size	No power calculation was undertaken. In genome-wide omics studies the larger the sample size, the greater the power and the greater the ability to detect genetic association with omic traits due to low frequency alleles. We therefore simply sought to maximise the available sample size. The total sample size of the transferrin glycosylation meta-analysis was 1907 (CROATIA-Korcula N = 948, Viking N = 959). The total sample size of the IgG glycosylation meta-analysis was 2037 (CROATIA-Korcula N = 951, Viking N = 1086).
Data exclusions	Quality control steps to remove SNPs prior to meta-analysis are described in the Methods. SNPs with MAC < 6 and/or displaying significant inter-cohort difference in allele frequency (allele frequency > +/- 0.3) were excluded.
Replication	We validated results obtained in the CROATIA-Korcula transferrin N-glycome GWAS by performing the same analysis in the independent VIKING cohort. We validated SMR-HEIDI significant findings using bi-directional Mendelian Randomisation followed by colocalisation, and, when available, an independent cohort, as detailed by Supplementary Table 18.
Randomization	The present study does not describe a randomization control trial, thus allocation and randomization were not relevant. Subjects analysed in this study were volunteers from the general population.
Blinding	The present study does not describe a blind RCT, thus it was not necessary to blind researchers. Subjects analysed in this study were volunteers from the general population. Allocation, treatment and randomization are not relevant to this study.

Reporting for specific materials, systems and methods

We require information from authors about some types of materials, experimental systems and methods used in many studies. Here, indicate whether each material, system or method listed is relevant to your study. If you are not sure if a list item applies to your research, read the appropriate section before selecting a response.

Materials & experimental systems

n/a	Involved in the study
☒	☐ Antibodies
☒	☐ Eukaryotic cell lines
☒	☐ Palaeontology and archaeology
☒	☐ Animals and other organisms
☐	☒ Human research participants
☒	☐ Clinical data
☒	☐ Dual use research of concern

Methods

n/a	Involved in the study
☒	☐ ChIP-seq
☒	☐ Flow cytometry
☒	☐ MRI-based neuroimaging

Human research participants

Policy information about studies involving human research participants

Population characteristics	The CROATIA-Korcula isolated population cohort includes samples of blood DNA, plasma and serum, anthropometric and physical measurements, information related to general health, medical history, lifestyle, and diet for ~3000 residents of the Croatian island of Korčula. The Viking Health Study - Shetland (VIKING) is a family-based, cross-sectional study that seeks to identify genetic factors influencing cardiovascular and other disease risk in the population isolate of the Shetland Isles in northern Scotland. Genetic diversity in this population is decreased compared to mainland Scotland, consistent with the high levels of endogamy. Fasting blood samples were collected and many health-related phenotypes and environmental exposures were measured in each individual. Covariate-relevant population characteristics of the human research participants (e.g. age, gender and genotypic information) are detailed in the Supplementary Table 17, for both cohorts. Participants were recruited among the general population and information about past and current diagnosis and treatments are not available nor relevant to the present study.
Recruitment	Participants were recruited among the general population. For VIKING cohort, participants were recruited between 2013 and 2015, most having at least three grandparents from Shetland.
Ethics oversight	All participants gave written informed consent. The CROATIA-Korcula study was approved by the Ethics Committee of the Medical School, University of Split (approval id: 2181-198-03-04/10-11-0008), the VIKING study was approved by the South East Scotland Research Ethics Committee, NHS Lothian (reference: 12/SS/0151).

Note that full information on the approval of the study protocol must also be provided in the manuscript.
